# Dogs’ reactions to motivations and emotions in conspecific and heterospecific vocalizations

**DOI:** 10.1038/s41598-026-46906-y

**Published:** 2026-04-01

**Authors:** Tamás Faragó, Lilla Kocsis, Beatrix Laczi, Irene Rojas Atares, Paula Pérez Fraga, Morgane Audiguier, Soufiane Bel Rhali, Katie E. Slocombe, Enikő Kubinyi, Attila Andics

**Affiliations:** 1 Neuroethology of Communication Lab, Department of Ethology, Eötvös Loránd University, Budapest, Hungary; 2 BARKS Lab, Department of Ethology, Eötvös Loránd University, Budapest, Hungary; 3 Department of Ethology, ELTE Eötvös Loránd University, Budapest, Hungary; 4 Department of Psychology, University of York, York, UK; 5 MTA-ELTE Lendület “Momentum” Companion Animal Research Group, Budapest, Hungary; 6 ELTE NAP Canine Brain Research Group, Budapest, Hungary

**Keywords:** Social evolution, Emotion, Animal behaviour, Behavioural ecology

## Abstract

Vocalizations convey information about both emotional valence (negative/positive) and motivational states (hostile/non-hostile). However, it remains unclear which of these dimensions primarily determines what the listener decodes from vocalizations as social messages, guiding their reaction (approach/withdraw). To test this question, we presented agonistic (negative|hostile), play/comfort (positive|non-hostile), and distress sounds (negative|non-hostile) to dogs. In Study 1, the motivational state encoded in conspecific calls better explained dogs’ reactions than emotional valence. Distress calls evoked faster approaches but slower withdrawal than agonistic calls, indicating that dogs primarily decode conspecific social messages based on the caller’s motivation. In Study 2, we tested cross-species decodability with chimpanzee and human calls and speech. Neither the callers’ motivational state nor emotional valence explained dogs’ reactions, which did not support predictions based on Morton’s rules and other proposed universal principles of emotion encoding. These findings suggest that the caller’s motivation may have been more significant in close-contact call evolution than emotions, and the mechanisms underlying the processing of conspecific vocalizations do not directly generalize to cross-species vocalizations. Consequently, decoding social messages from vocalizations may rely less on universal rules than previously thought.

**Supplementary Information:**

The online version contains supplementary material available at 10.1038/s41598-026-46906-y.

## Introduction

The evolution of inner-state communication is driven by two competing selective pressures: the interests of the signaler and the receiver^1,2^. The signaler aims to change receiver behaviors for its own benefit; this promotes manipulative signaling^3^. In contrast, the receiver aims to predict the signaler’s behavior reliably and to react adaptively; this promotes decoding cues that are encoded due to different constraints^4^. Therefore, signals are selected to influence receivers, whereas cues arise as honest by-products of constraints and may be exploited by listeners. The effectiveness of communication during social interactions depends heavily on the encoded information the receiver extracts, as this determines the decoded social message and thus, ultimately, their reactions. Vocalizations play a crucial role in organizing these social interactions, carrying a wide variety of information^5^, including about dynamically changing inner states^6^, mostly encoded in short-range calls that are less constrained by environmental distortion and, crucially, have a specific, well-defined aim, the actual interaction partner. Evidence that encoded cues take priority over signals in affecting the decoded social message thus would suggest that listener interest had a greater role in short-range call evolution, while, conversely, the priority of signals over cues would reveal a greater role of the caller’s interest^4^.

**Fig. 1:**
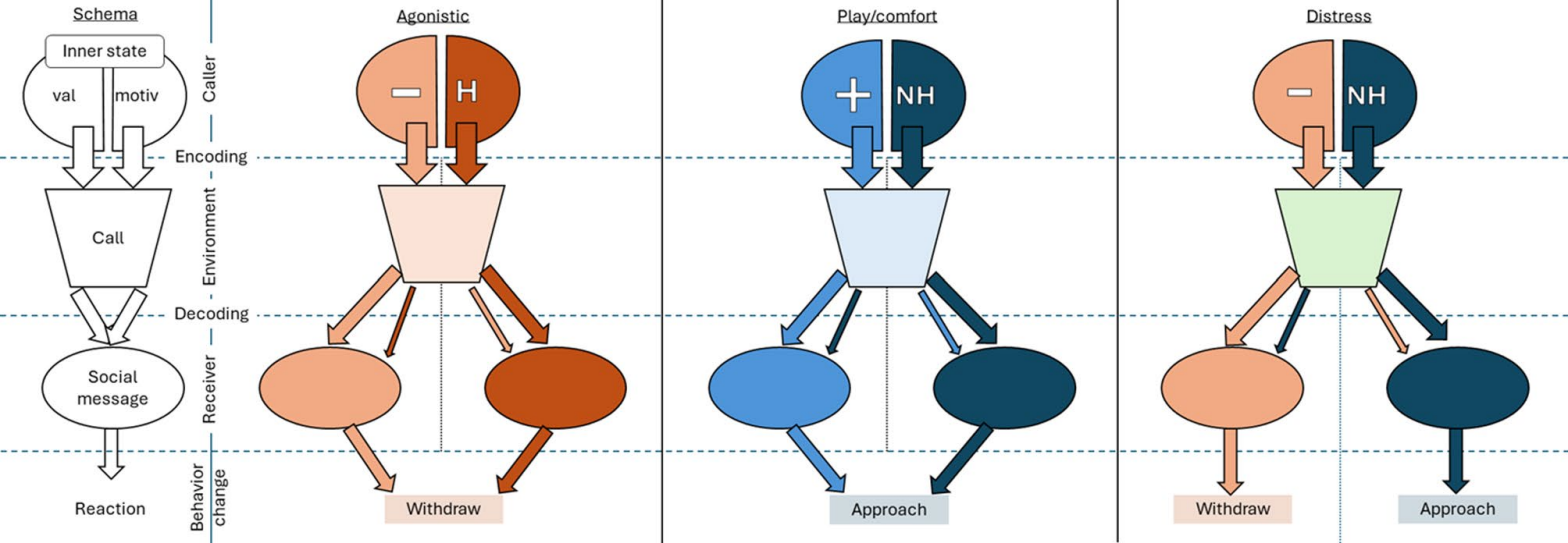
The overview of the theoretical framework and the expected differences across three social contexts. Within each context panel, the left side shows when emotional cues take priority in affecting the social message, while the right side shows the priority of motivational state. In the case of distress calls, potentially both might affect the social message, leading to opposite behavioral outcomes. Abbreviations: val: valence state, motiv: motivation state, +: positive valence, -: negative valence, H: hostile, NH: non-hostile.

Both emotions and motivations have a significant role in organizing social behavior and thus, if extracted from vocalizations, either or both can potentially be decoded as part of the social message (Fig. 1). Emotions, simply put, are specialized states of the nervous system that enhance the chance to respond adaptively to external events^7^. A key dimension of these states is valence (positive vs. negative)^8^. Through innervation of the vocal apparatus, emotional states affect different aspects of voice production, creating emotion cues in the produced sounds following well-described acoustic rules^6^. Although these rules are more clear-cut in the case of arousal (the other main dimension of emotional states), general acoustic associations with valence were found across species: e.g., positive sounds have shorter length and lower, less variable pitch than negative sounds^6^. Emotional valence reflects the hedonistic value of the context and is thus advantageous for the listener to decode. Motivation can be considered the inner driving force that directs the individual’s action, and different motivational states manifest in different behavioral outcomes^9^. Morton’s Motivation-Structural (MS) rules suggest that “hostile motivation” leading to agonistic behavior is linked with low pitch, harshness and broad spectrum, while the lack of hostility (e.g., fear/submission) is associated with high pitch and tonality^10^. Additionally, August & Anderson (1987) extended the MS rules^11^, differentiating positive and negative calls within the non-hostile category, suggesting that affiliative calls are soft, rhythmic, low-pitched, and noisy. Such vocal signaling of motivational states before behavioral manifestation enables the caller to change listener behavior, but only if listeners decode and use them. Emotions and motivations are interlinked in the caller^12^. As different motivational states can be present in an individual potentially simultaneously, some mechanisms are necessary to select among these to organize behavioral reactions. Emotions, especially valence, through adding hedonistic values to these motivational states, help in prioritizing among them and ultimately determining the decision which will control the behavior. On the other hand, the resulting behaviors through affecting the environment and the reactions of the interaction partners can alter the caller’s emotional state, creating a feedback loop between emotions and motivations. Still, both are encoded in the acoustic structure of the produced calls. Though the rules concerning this encoding are well described^6^, it is still unclear whether cues carrying honest emotional state information or signals of motivation encoding potentially manipulative information aimed to influence the behavior of the receiver are primarily decoded as the social message by the listeners, ultimately affecting their approach or avoidance reactions during interactions.

In terrestrial vertebrates, both the mechanisms of voice production and the neural circuitry involved in emotions and motivations are homologous^4,6,13^. Although these homologies lead to shared encoding rules and thus, in principle, make acoustic correlates of inner states suitable for cross-species communication (for a recent review see^14^, evidence for efficient decoding of social messages from the vocalizations of another species is mixed^15^. Reports supporting cross-species decodability of emotional valence are mainly based on humans assessing non-human sounds (e.g., dogs^16–18^, pigs^19^, apes^20,21^, horses^22^ and a range of ungulates^23^. There are indications of negative and positive valence discrimination in both conspecific and heterospecific vocalizations, mostly of human sounds in dogs^24^, horses^25,26^ and pigs^25^. In contrast, other studies found that humans failed to decode emotional valence from the calls of unfamiliar^27^ and/or phylogenetically distant species^28^. Regarding motivational states, while there is ample evidence that their encoding follows Morton’s rules based on the contextual usage of call types (e.g., coati^29^, meerkat^30^, tiger^31^, African wild dog^32^, howler monkey^33^, giant otter^34^, humpback whale^35,36^, and bats^37^, on the listeners’ side, comparatively little is known. There is some evidence that the encoded motivational state might indeed be decoded by conspecifics (e.g., deer^38^, rock hyrax^39^; still, no study has directly explored cross-species decoding of motivational state. One study investigated cross-species processing of distress calls in mule deer and white-tailed deer in a setting that could be interpreted as a test of motivational state decoding. However, the use of a particular mother-offspring context limits the generalizability of its findings. Nonetheless, this study reported that although cross-species decodability may have been present in some instances, it was dependent on the acoustic overlap between the tested heterospecific and conspecific calls^40^. Taken together, these reports on processing either emotional valence or motivational states, there is at present no consensus on the cross-species decodability of inner states.

Here, to assess the relative contributions of vocal emotional valence and motivational state information to the decoded social message, we used vocalizations from three different social situations, characterized by different combinations of caller emotional valence and motivational state (Table 1). Distress contexts have a negative hedonistic value, reflected in callers’ emotions (negative valence), but also result in a motivation to attract interaction partners (non-hostile motivation), leading to potentially opposing social messages, and making it possible to disentangle their contribution in affecting listeners’ reactions^41^. Play/comfort contexts both have a positive hedonistic value for the caller (positive valence) and evoke motivation to attract and maintain social proximity with an interaction partner (non-hostile motivation) to sustain the interaction. In contrast, agonistic contexts are clearly linked to negative hedonistic value, resulting in negative valence in the caller and a motivation to repel or chase away the interaction partner (hostile motivation).

**Table 1:** The details of the sub-groups, defined by the context and the call species/type combinations.

Context					Source
		Distress	Agonistic	Play/comfort	
	*Emotional valence*	negative	negative	positive	
	*Motivation*	non-hostile	hostile	non-hostile	
Species	*Dog*	separation whines	food guarding growls	play/greeting/petting grunts, pants, growls, moans	Family Dog Project database^16,42–45^
	*Human nonverbal*	sad cries, loss	rage roars, losing, being cheated	amused laughs, play contexts	Anikin & Persson database^46^
	*Speech*	sad intonation	angry intonation	happy intonation	RAVDESS corpus^47^
	*Chimpanzee*	separation whimper	waa-bark from conflict	laughter, play, wrestle, tickle	Gombe database^48^ & Katie Slocombe^20^
		Call types and context details			

First, in Study 1, we played back to dogs (*Canis familiaris*, *N* = 59) conspecific vocalizations recorded in these three different contexts in three groups. Then, in Study 2, to test cross-species decodability and compare it to the pattern for conspecific decoding, we played back to different groups of dogs (*N* = 178) heterospecific vocalizations (human and chimpanzee) and speech recorded from different social contexts that presumably evoke matching inner states as the contexts used in Study 1. Dogs, selected for cognitive abilities during domestication, such as elevated social attention^49^, are good models for such comparative studies as they are deeply immersed in a heterospecific vocal environment and gain considerable experience with human vocalizations and speech^50^, providing a unique possibility to test their reactions to heterospecific sounds on two different levels of social relevance: human sounds being highly relevant for dogs and phylogenetically close chimpanzee (*Pan troglodytes*) calls being unknown and socially irrelevant for dogs. Additionally, in the case of human sounds, we can test both biologically rooted non-verbal emotion expressions and speech intonation, both known to have importance in dog-human communication^51,52^.

We predicted (see Fig. 1) that if the listeners’ decoded social messages are primarily determined by (1) the caller’s motivational states, non-hostile sounds (distress and play/comfort) would be more likely to evoke an approach rather than hostile ones (agonistic), which would evoke withdrawal. More specifically, at the level of contrasting distress and agonistic contexts, we predicted that if motivational state determines dogs’ reactions, then distress sounds will elicit a more likely, faster approach and a less likely, slower withdrawal than agonistic sounds. In contrast, if the listeners’ decoded social messages are primarily determined by (2) the caller’s emotional valence, then positive sounds (play/comfort) will evoke an approach more likely than negative ones (distress and agonistic), which are expected to evoke withdrawal more likely. Specifically, at the same contrast, if emotional valence determines the reactions, distress sounds will elicit more likely and faster withdrawal and less likely and slower approach than play/comfort sounds. Alternatively, if (3) neither emotional valence nor motivational state is decoded by the listener, then type and latency of the approach-withdrawal reactions will be independent of the sound context. Furthermore, if Morton’s rules are at play, we expect prediction 1 to be accurate for heterospecific sounds as well, whereas when general rules of vocal emotion encoding are at play, we expect prediction 2 to be accurate in Study 2.

## Results

In a between-subjects design, each participating dog was presented with one call condition only (either distress, agonistic or play/comfort) from a hidden speaker in our laboratory. Across both studies, the groups defined by the call species/type and the context were balanced for sex and age (see Supplemental S7-8 Fig and S7 Table). Each dog heard one unique, one-minute-long, randomly assembled sequence containing all samples from our collected sound pool of the given call species/type and context. First, we analyzed the type of initial reactions (approach, withdrawal, or no reaction), to test our main hypotheses 1 and 2 about motivational state and emotional valence effects. As our context composition does not allow us to formally test for interaction between valence and motivation due to a missing hostile-positive context, for a more detailed analysis, we used the contexts (agonistic, distress and play) as a factor in the analysis of the latencies of the initial reactions to these playbacks (for details, see the Methods section below). Here, we also included the dogs’ demographic features (sex, age) in the models. Our rationale for this different model composition was to keep the main hypothesis-testing models as simple as possible, while allowing latencies to provide more fine-grained insight (not just the type of reaction, but also its timing can be tested) for these secondary models.

### Study 1: Reactions to conspecific vocalizations

**Fig. 2:**
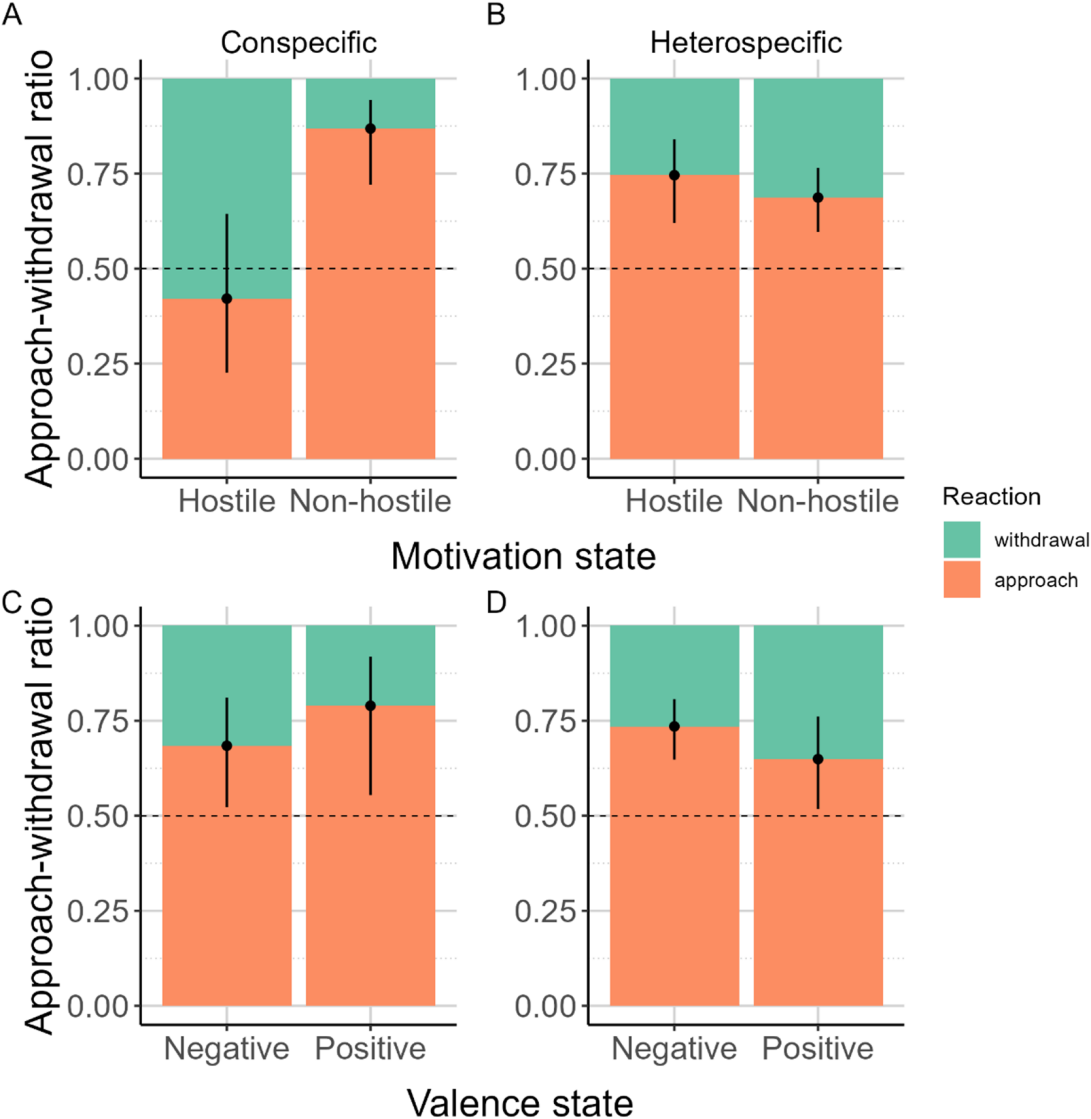
The ratio of the initial reactions to con- (A, C) and heterospecific (B, D) sounds, depending on the callers’ motivational or valence state. The dots and whiskers show the binomial model estimates and 95% confidence intervals.

First, to test whether the motivational state (hostile or non-hostile) or the emotional valence (positive or negative) of the conspecific vocalizations affects the decoding of the social message by the dogs, we analyzed their initial reactions (approach or withdrawal), comparing binomial generalized linear models including either the motivational state or the valence of the calls or both. We found that the model containing motivational state alone explains the responses best (see Table 2 and S1 Fig) and, accordingly, motivational state had a significant effect (LR Test: χ^2^(1) = 12.216; *p* < 0.001): dog vocalizations with non-hostile motivation (distress and play/comfort calls) were nine times more likely to evoke an approach reaction than hostile, agonistic calls (o.r.[95%CI] = 9.075[2.580–36.548.580.548]; z = 3.302; *p* < 0.001; Fig. 2A). Although slightly more dogs reacted with withdrawal than approach when hearing agonistic growls, the approach-withdrawal ratio did not differ significantly from chance based on the estimated probabilities. While based on AICc values, the model including both Motivation and Valence differs negligibly from the Motivation only model, Valence had no significant effect (LR Test: χ^2^(1) = 2.201; *p* = 0.138, Fig. 2C) while Motivation had (LR Test: χ^2^(1) = 13.699; *p* < 0.001; for model details see Suppl. Table S8).

**Table 2:** The results of the model inference for the within-species binomial models.

model terms	aicc	weights
~ Motivation	59.68	0.501
~ Motivation + Valence	59.71	0.494
Null model	69.75	0.003
~ Valence	71.18	0.002

Next, to get a more detailed view of possible context (distress, agonistic, and play/comfort) and individual (sex and age) effects and analyze the dynamics of the two reaction types separately, we compared the latency of the first approach and withdrawal reactions using Cox proportional hazards models. This analysis uses the toolbox of survival modelling for latency data which, on one hand is considered to be more appropriate than applying linear models or non-parametric techniques as it takes into account the cases where the observed reaction did not occur (censored cases) within the observation time (maximum latency), but treats them differently from the regular cases avoiding the inflation of the large latency values that potentially distorts the distribution rendering other more common analysis ways inappropriate^53^. On the other hand, this analysis provides insight into the temporal dynamics of the observed reaction by handling occurrence and latency data in a combined way, revealing the factors affecting both the speed and the likelihood of reactions. For approach latencies, when the dog showed no approach or withdrew first, we considered it a censored case, and for withdrawal, vice versa. Call context, subject sex, and age were included as main effects in both initial models, while all possible two-way interactions were included only in the approach latency model, as initial withdrawal was a rare event in the case of distress calls, and thus models with interaction terms failed to converge due to an insufficient number of cases. To identify the most parsimonious model, we applied exhaustive model inference again (S1-2 Table and S2-3 Fig).

**Fig. 3:**
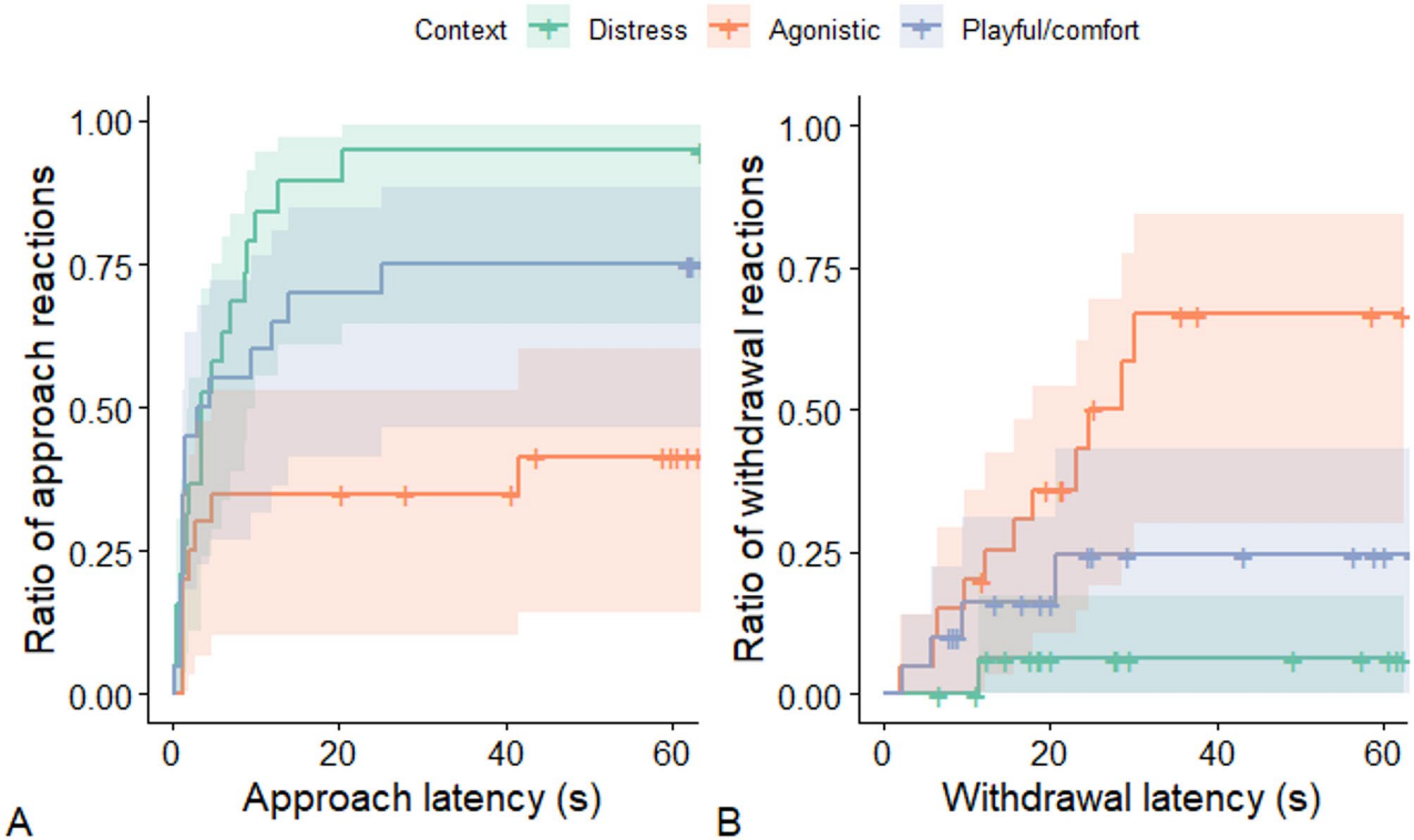
The change in the cumulative ratio of individuals over time reacting to the playbacks, depending on the sound context. The steeper the lines, the shorter the latencies, the higher they are, the higher the odds of showing the behavior. Shading around the lines shows the 95% confidence interval. Cross-ticks on the lines are showing censored cases.

For approach latencies, the most parsimonious model included only the context, and it had a significant effect (LR test: χ^2^(2) = 11.392; *p* = 0.003). Tukey post-hoc tests (Fig. 3A) revealed that dogs were quicker and more likely to approach distress calls (exp(β)[95%CI] = 0.258[0.093–0.715]; z=−3.114; *p* = 0.005) and also tended to be slightly quicker and more likely to approach play/comfort calls (exp(β)[95%CI] = 0.375[0.134–1.049]; z=−2.234; *p* = 0.066) than agonistic calls, while approach reactions to distress and play/comfort calls did not differ (exp(β)[95%CI] = 1.455[0.632–3.351]; z = 1.055; *p* = 0.542). Similarly, withdrawal latencies were also most affected by context (LR test: χ^2^(2) = 10.173; *p* = 0.006). According to the post-hoc test (Fig. 3B), agonistic calls tended to evoke withdrawal slightly more likely and faster than distress calls (exp(β)[95%CI] = 10.936[0.945–126.5]; z = 2.290; *p* = 0.057), while withdrawal reactions to agonistic calls and play/comfort calls did not differ (exp(β)[95%CI] = 2.702[0.687–10.6]; z = 1.701; *p* = 0.205). There was no difference between withdrawal reactions to distress and play/comfort calls (exp(β)[95%CI] = 0.247[0.018–3.4.018.4]; z=−1.250; *p* = 0.424). In both the approach and withdrawal latency models, neither age nor sex of the dog had any main or interaction effect.

## Study 2: reactions to heterospecific sounds

For heterospecific sounds, we found no model that outperformed the null model, suggesting that neither sound category, the caller’s motivational state, nor its emotional valence explained the dogs’ initial reactions (Fig. 2B-D; Table 3, S4 Fig, for model details see Suppl. Table S9).

**Table 3:** The results of the model inference for the cross-species binomial models.

model terms	aicc	weights
Null model	212.53	0.32
~ 1 + Valence	213.23	0.22
~ 1 + Motivation	213.92	0.16
~ 1 + Motivation + Valence	215.24	0.08
~ 1 + Category	215.51	0.07
~ 1 + Category + Valence	216.27	0.05
~ 1 + Category + Motivation	216.88	0.04
~ 1 + Category + Valence + Valence: Category	218.15	0.02
~ 1 + Category + Motivation + Valence	218.29	0.02
~ 1 + Category + Motivation + Motivation: Category	218.72	0.01
~ 1 + Category + Motivation + Valence + Motivation: Category	220.12	0.01
~ 1 + Category + Motivation + Valence + Valence: Category	220.22	0.01
~ 1 + Category + Motivation + Valence + Motivation: Category + Valence: Category	223.34	0.00

**Fig. 4:**
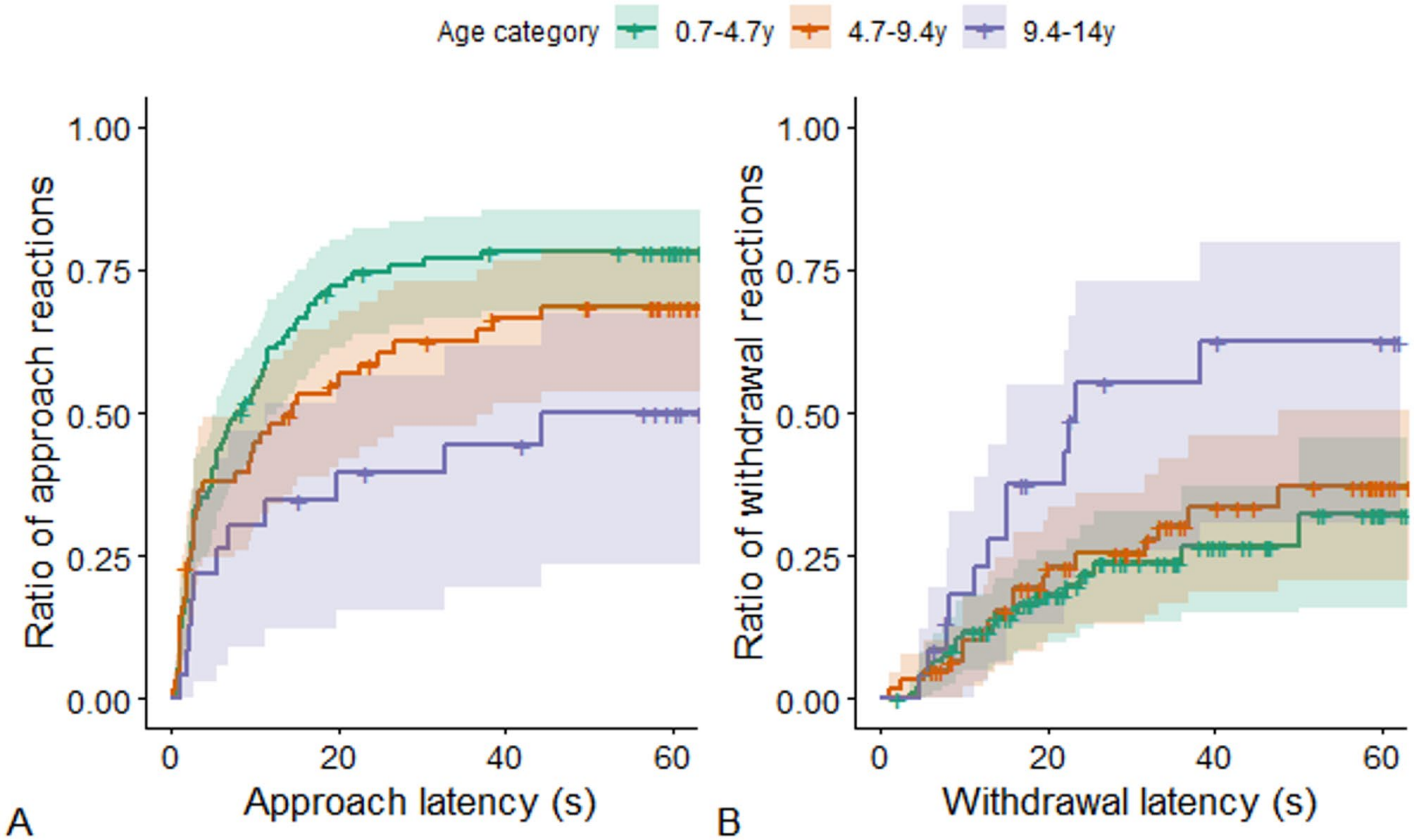
The change in the cumulative ratio of individuals over time reacting to the playbacks, depending on the individuals’ age. Note that the age was a continuous variable in the models, and 33–66% quantile categorization was used only for visualization’s sake. The steeper the lines, the shorter the latencies, the higher they are, the higher the odds of showing the behavior. Shading around the lines shows the 95% confidence interval.﻿ Cross-ticks on the lines are showing censored cases.

Following the analysis steps in Study 1, we next tested whether the call context, the subjects’ sex and age, and additionally the sound category (chimpanzee calls, human nonverbal emotional calls, and speech) or their interactions affected the latency of the initial approach or withdrawal reactions. The most parsimonious models (S3-4 Table, S5 Fig) included age, sex, context, and the sex-context interaction for approach latencies and age for withdrawal latencies (S5-6 Table, S6 Fig). These models showed that dogs’ age had a significant main effect on both approach (LR test: χ^2^(1) = 9.001; *p* = 0.003; Fig. 4A) and withdrawal (LR test: χ^2^(1) = 5.663; *p* = 0.017; Fig. 4B). Independent of context and sound category, older dogs were less likely and slower to approach (exp(β)[95%CI] = 0.923[0.874–0.974]; z=−2.907) and more likely and faster to withdraw (exp(β)[95%CI] = 1.103[1.019–1.194]; z=−2.419) than younger dogs.

**Fig. 5:**
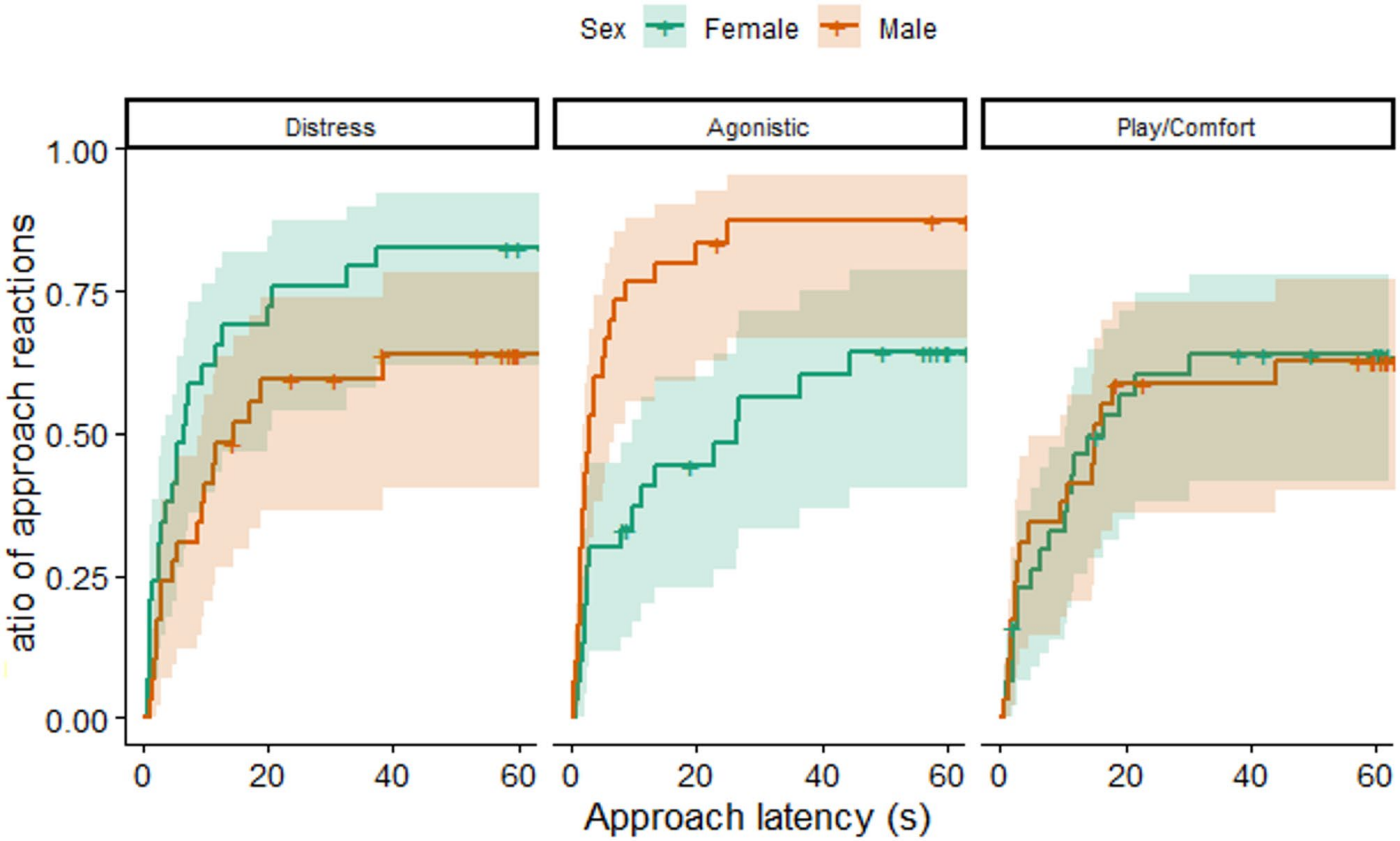
The change in the cumulative ratio of individuals over time reacting with an approach to the playbacks, depending on the sound context and sex. The steeper the lines, the shorter the latencies, the higher they are, the higher the odds of showing the behavior. Shading around the lines shows the 95% confidence interval. ﻿Cross-ticks on the lines are showing censored cases.

Regarding approach latencies, we also found an interaction effect of sex and context (LR test: χ^2^(2) = 12.594; *p* = 0.002; Fig. 5). The post hoc test showed sex difference in the reactions to agonistic sounds: independently from the sound category (chimpanzee, human vocalizations or human speech), female dogs were less likely and slower to approach these sounds (exp(β)[95%CI] = 0.374[0.204–0.685]; z=−3.184; *p* = 0.002). We also found an opposite trend in the case of the distress context: females tended to be more likely and faster to approach distress sounds (exp(β)[95%CI] = 1.733[0.940–3.196]; z = 1.762; *p* = 0.078). There was no sex difference in the approach latencies of play/comfort sounds (exp(β)[95%CI] = 0.959[0.503–1.827]; z=−0.129; *p* = 0.898).

## Discussion

Here, we provided a large, unique dataset of playback recordings comparing dogs’ reactions to sounds from conspecifics, humans, and chimpanzees from different social contexts. Study 1 revealed that, for conspecific calls, the callers’ motivational state better explained the dogs’ initial reactions than the callers’ emotional valence. More specifically, distress or playful/comfort contexts were more likely to elicit approach reactions from dogs than agonistic growls, and they also approached calls from distress or playful/comfort contexts faster than those from agonistic contexts. This pattern suggested that, for conspecific calls, listeners’ adaptive responses were driven by the social message decoded from callers’ motivational state rather than the emotional valence. In contrast, Study 2 showed that for heterospecific sounds, neither motivational state nor emotional valence explained dogs’ initial reactions. For human and chimpanzee sounds, dogs did not appear to use either the presumably encoded motivational or emotional state of the caller to inform their behavioral reaction. Instead, they were affected by individual-specific features such as listener age or sex. Together, these findings provide novel evidence that behavioral reactions to con- and heterospecific sounds follow different organizing principles in dogs.

Different reactions to conspecific calls from markedly different social contexts provide evidence that the caller’s motivational state is efficiently decoded as the social message, ultimately affecting the behavior adaptively. Obviously, failing to respond appropriately upon hearing hostile, agonistic calls is more costly than misinterpreting non-hostile ones. This cost difference might facilitate greater sensitivity to threat signals and cues, as reflected in dogs’ slower approach and faster withdrawal to threatening calls. Still, it is insufficient to explain the markedly faster and more likely approach reactions to the distress whines. Specifically, for threatening calls, our results extend earlier findings that dogs react differently to conspecific food-guarding vs. play growls in a food competition context^43^ by showing that food-guarding growls are also effective in a more neutral setting and elicit a response which is clearly different from that to play calls. For distress calls, the more likely and faster approach and the lower occurrence of withdrawal are in line with an earlier study^54^. In that study, dogs were found to spend more time close to a speaker when hearing separation whines than control sounds and tended to approach and show affiliative behaviors to a known conspecific after these playbacks. Thus, it seems that dogs generally tend to approach conspecific distress calls.

Combining reactions to conspecific calls across the three different contexts, especially the approach reactions to distress calls, provides evidence that the caller’s motivational state has priority over the caller’s emotional state in determining the decoded social message. While the distinct reactions to calls from different contexts are also in line with findings in other species, in most of these earlier playback studies, either only one specific call type’s communicative role was explored^30,38,55^, or the emotional and the motivational state was not appropriately separated (e.g., because both agonistic and distress calls were used to represent negative emotional valence^25^. Consequently, based on these previous results, we cannot decipher what actually affects the measured reactions and, ultimately, the contribution of signaler and receiver interests to short-distance call evolution. However, by contrasting the lack of emotional valence effect with the effect of motivational state in explaining the approach-withdrawal patterns, our results support the idea that signalers’ interest in affecting the behavior of the receiver may have played a greater role than the listeners’ interest in reading emotional cues in the evolution of short-distance calls. These findings thus provide experimental support for the role of Morton’s Motivation-Structural rules in call evolution.

The importance of motivational state decoding in social interactions does not mean that emotional state (both valence and the other commonly used dimension, arousal) cues are not perceived or that they do not contribute to social message decoding. On the one hand, dogs indeed process valence in conspecific calls^56^; thus, it seems unlikely that, in our particular case, they did not perceive emotional cues encoded in the playbacks. On the other hand, dogs’ ability to read emotional cues is evidenced by reports of emotion contagion and reconciliatory behaviors^57^. Although an emotional cue-reading-based account of social message decoding would predict avoidance of separation whines, emotion contagion might still provide an alternative explanation to motivational state decoding for why dogs approach rather than avoid separation whines (instead of avoiding them). Notably, emotion contagion may elicit such empathy-like behaviors without the need for cognitively understanding the distress of others and thus approach to provide comfort to alleviate their distress. A more parsimonious explanation might be ‘selfish’ comfort-offering where individuals lower their own distress by reducing the source of the emotional contagion (see helping rats^58^, or in a cross-species context, even predatory exploration, as recently found in crocodiles^59,60^. Nevertheless, while emotion contagion might thus explain reactions to distress calls, the same argument cannot be applied to agonistic growls. As agonistic growls also have negative valence, emotion contagion would suggest a similar reaction to distress calls, but this is not the case. Thus, the argument that dogs decode social messages from the encoded motivation (hostile vs. non-hostile) despite the also perceived negative valence and react accordingly seems more plausible. A further, more direct argument for dogs’ ignorance of emotional valence cues for social messages could be the finding of no response difference to positive but hostile-motivation sounds (e.g., aggressive laughter in gloating, schadenfreude, ridiculing someone, etc.^61^ and negative but hostile motivation sounds. However, to our knowledge such positive, hostile-motivation sounds, have not been described in canids. In other species, one example of such vocalizations can be the post-conflict acoustic signals (PCAS) produced by winners, a.k.a. triumph vocalizations (see for review^62^ that are thought to function to decrease the likelihood of future conflicts, thus constituting positive|hostile calls^63^. Comparing these with pre-conflict threat calls might open up an interesting research avenue in the evolution of vocal communication.

It needs to be mentioned here that, although in our study the playback context was constant across groups in order to keep the subjects’ inner state standardized and as similar as possible, in general, the receivers’ inner state might influence how the decoded social message affects their reactions. This can lead to individual variability and potentially the same individual e.g., with a different hunger level might react differently to the same playback. In the wild, sub-optimal or even maladaptive reactions might happen in certain extreme cases, like a high motivation state of the receiver might override the decoded social message and drive a hungry individual to ignore the agonistic vocalizations of the caller in a food competition context. However, such edge cases might play a less likely role in call evolution due to their rarity and the elevated costs they impose on both parties. Also, certain call types with specialized functionality might complicate the picture, too. While short-distance calls have an intended, dedicated aim, the receiver whose behavior the caller tries to manipulate in the interaction, long-distance calls broadcast information in a less directed way, opening the possibility of a wide range of receivers with various social contexts and motivations affecting how they decode information from these calls. For example, territorial barks of wild canids (and, supposedly, dogs’ too) are considered to be mobbing signals^64^. These calls, while clearly linked to a negatively valenced inner state, aim to elicit different reactions depending on the receiver. While hearing the bark of a packmate within territory should evoke an approach reaction for joint defense, in contrast, if the listener is the intruder, the decoded social message from the same vocalization should evoke withdrawal. This suggests that in medium/long-range calls, where the audience is not controlled, the decoded social message can vary, while in short-range calls, where the caller aims the call towards a specific receiver, the message must be much clearer, thus these calls are ideal for studying the evolution of information encoding.

Finding no effect of context on the response patterns to heterospecific sounds, however, suggested that the role of internal-state-encoding rules in governing listener reactions is less evident than previously assumed. One reason for this may be that call structure evolution might have taken largely different trajectories in different species, pushing similar-context call types into partially distinct niches in the acoustic space^65^. Although there is ample evidence that similar anatomical and physiological constraints across species govern encoding rules^66^, it is possible that the acoustic niches of evolutionarily more distant species’ calls do not have a sufficient overlap for these rules to be efficiently exploited during decoding. In support, in deer^40^ and bats^67^ for example, a decrease in this overlap leads to less efficient decoding of heterospecific calls. Conversely, several studies reported some form of heterospecific emotion discrimination. Notably, however, most of these cases come from domesticated species reacting to human sounds^26,68–70^. These sounds are highly relevant to the animals as humans interact with them daily. Relevance, especially ecological relevance, is known to affect heterospecific reactions in the special case of alarm calls^71–73^ and prey-predator interactions^59,60^. Still, the fact that dogs did not show the approach/withdrawal behavioral reactions expected based on either valence or motivation encoded in human sounds in the present study, despite humans being highly relevant to them in contrast with chimpanzees, suggests that relevance alone may not be sufficient to make social messages efficiently affect behavior between evolutionarily distant mammals. This inefficiency potentially leads to behaviorally unreliable social message decoding from heterospecific sounds, thus other, individual-specific factors might have more influence on the reactions independently of the encoded information in the calls. Still, looking at more subtle behavior changes, physiological and neurological reactions might still reveal the perception and decoding, even in the lack of clear approach-avoidance reactions.

In line with this, our finding seemingly contradicts previous reports that dogs excel at reading human vocal emotions^24,56,70,74,75^, but it is also possible that other factors, like sex-dependent personality differences, overshadow the effect of social messages on dogs’ reactions. Indeed, the fact that male dogs approached agonistic sounds more than females might be due to males being bolder or females being more sensitive to social information. Supporting the male boldness effect, Plutchik reported that female dogs have a stronger tendency to avoid novel objects, whereas males are more likely to approach them^76^. Similar patterns can be found in various species, showing males’ tendency to approach con- and heterospecific calls regardless of their encoded social message (e.g., collared pika^77^, chimpanzees^78^, white rhinoceros^79^, but see macaque boom calls where males avoid but females approach the sound source^80^. All these and our results might relate to males’ higher competitiveness across species (for a review, see^81^. Alternatively, but not exclusively, elevated female sensitivity in the social domain might also explain the results of males’ seemingly sub-adaptive response. There are indications of such female attentional or processing biases in dogs in the domain of physical cognition^82^, olfactory processing of emotional odors^83^ and also in general sociability^84^. In humans, numerous reports indicate that females have an advantage over males in emotion processing^85^, which may be linked to the processing of others’ motivational states and intentions. Although sex differences in sociability strongly depend on the social structure of the species^86^, in dogs (in contrast with wolves), females have sole responsibility for raising the pups; thus, they might be under more substantial selective pressure to be more socially sensitive than males. However, there is no evidence of this female advantage in play/comfort sounds, and aside from the lack of approach to agonistic sounds, only a slight trend suggests that females are more likely to approach in case of distress sounds. Thus, sex-dependent boldness might be more likely in play in this case.

The finding that age, independent of all other factors, affected response patterns to heterospecific sounds might be attributed to several reasons. One possibility is a simple age-related motor or sensory decline resulting in slower movements and slower reaction times. However, this is unlikely in our case, as we also found that older dogs were quicker to withdraw. A second possibility is an age-related shift in valence processing, specifically a negativity bias, namely, perceiving the same stimuli as more negative with age. However, this would contradict previous findings suggesting age-related positivity, rather than negativity, bias in both humans^87,88^ and dogs^89^. Consequently, we propose that older dogs’ faster withdrawal, which in our case can also be interpreted as approaching the owner more quickly, is probably due to greater reliance on the owner through social referencing or the ‘safe-haven’ effect. On the one hand, older dogs may generally have slower emotional or social cognitive processing abilities and thus rely more on social referencing their owners^90^, that is, trying to gain information from their reactions in ambiguous or scary situations^91^. Furthermore, it is known that with ageing, dogs cope less effectively with distress and depend more on their owners, which is reflected in stronger attachment^92^, including the ‘safe-haven’ effect, that is, seeking the owner’s proximity to alleviate stress caused by a strange situation^93^. In our case, as the owners were asked to remain passive and avoid interaction with their dogs during the playbacks, the ‘safe-haven’ effect is more plausible, but further studies are needed to differentiate between these explanations.

One potential limitation is that other sensory modalities or metacommunicative signals did not accompany the playback stimuli as they would in natural contexts, and, in some cases, this may have led to misinterpretations. Specifically, dogs during play use several behavior elements that are parts of agonistic interactions or are used as hostile signals, but also a particular visual metacommunicative signal, the play bow, that ensures correct interpretations^94^. Indeed, this might explain that, unlike the clear-cut difference in reaction latencies to agonistic and distress conspecific calls, both approach and withdrawal latencies to play/comfort calls show an ambivalent, intermediate reaction. Possibly due to the lack of visual metacommunicative signals, some dogs might have perceived play growls as ambivalent signals evoking avoidance, especially as these calls were also found to indicate larger-than-real body size, which dogs also perceive accordingly^45^. A potential solution would be to represent a positive, non-hostile inner state using comfort calls that are less ambiguous in the absence of contextual information. However, this lack of metacommunicative signals did not affect most other responses. Moreover, using audio playbacks alone has obvious advantages: we can ensure that the measured reactions are influenced solely by the sounds themselves.

Another limitation might be differences in inherent arousal across contexts. This difference might pose an issue because arousal encoded independently from valence has been shown to be perceived^95,96^ in vocalizations and to affect behavioral reactions: specifically, higher arousal evokes stronger attention and vigilance^97^ or a faster approach^60^. Indeed, in our study, arousal is expected to vary systematically across contexts, as both distress and agonistic calls are considered high-arousal calls, whereas play/comfort calls are associated with lower arousal level. Based on this, however, one would expect weaker reactions to the play/comfort calls, but this pattern was not observed in either con- or heterospecific sounds. This suggests that arousal did not contribute significantly to the differences across contexts in dogs’ behavioral reactions.

In conclusion, our results provide a demonstration of the primacy of encoded motivational states in short-distance conspecific calls in determining listener behavior. This points to the direction that signaling motivational state may have played a more significant role in such directed, close-contact call evolution than cues of emotional states. Moreover, despite the presence of general acoustic encoding rules of both motivation and emotion, heterospecific decoding of social messages may be less universal than previously thought. This emphasizes the need for further comparative playback studies to disentangle the contribution of different encoded information in determining the social message listeners decode from vocalizations.

## Materials and methods

### Experimental Design

Apart from the playback materials, Studies 1 and 2 were conducted, and the data were analyzed the same way. Thus, we report their methods together in this section.

### Subjects

We tested adult family dogs (N_original_=277, N_actual_=237; ♀=121; mean age ± SD = 5.13 ± 3.26) with healthy hearing according to the owners and recruited through social media. Forty dogs (14% of the full sample, which is not unusually high for similar experiments) had to be excluded due to technical errors (camera or sound system failure) or significant deviations from the protocol caused by experimenter or owner errors. Subjects were distributed in a balanced manner across the playback species and context subgroups (Table 4), while accounting for the age and sex of the dog (see S7-8 Fig) in a between-subject design to avoid the potential carry-over effects from the first-heard sound types. The N per group ranged from 18 to 21. To confirm that there are no age differences across the playback species/type and context subgroups, we ran a linear model with subject age as the response variable and the main effects and the two-way interaction between context and species/type, with subjects from Studies 1 and 2 pooled. The F-test based model comparison showed no significant interaction effect (F(6) = 1.061; *p* = 0.387). For detailed model results, see S7 Table. Grouped chi-square tests confirmed that there were no imbalances across subgroups regarding the subject sex (S8 Fig).

**Table 4:** Subject numbers across the subgroups.

Context				
		Distress	Agonistic	Play/comfort
**Conspecific**	*Dog*	19	20	20
**Heterospecific**	*Human nonverbal*	21	20	20
	*Speech*	18	21	20
	*Chimpanzee*	19	19	20

## Ethical note

All participation was voluntary; owners signed an informed consent form before the tests, and they could terminate the tests at any time if they felt it was too stressful or scary for their dogs (there were no such cases). We confirm that the procedures were in accordance with relevant ethical guidelines and regulations and that they followed the ARRIVE guidelines. Ethical approval was obtained through the National Animal Experimentation Ethics Committee of Hungary (PEI/001/1056-4/2015).

## Sound material

Sound material for the playback sessions was collected from various sources from multiple individuals (Table 1).

In Study 1, dog vocalizations were collected from the sound database of the Family Dog Project. As distress calls (N_sound_=10), we chose adult separation whines recorded during separation from the owner in the laboratory^42^, as these calls can be considered as contact calls associated with stress experienced in the absence of the attachment figure (the owner)^42,98^ and thus can be analogous with infant separation cries. Separation whines are produced in bouts containing high-pitched and relatively clean calls with varying lengths, but also often containing non-linear phenomena^42,99^ and have a function of raising the attention of and reinstating contact with the owner, but they might also be able to evoke empathy-like reactions from other dogs^54^. For agonistic calls (N_sound_=10), we chose food-guarding growls recorded in a situation where the focal dog protects a bone from a potential competitor^43,44^. These agonistic growls are long, low-pitched and harsh, are rated highly aggressive by humans^18^ and have a potential repellent effect on other dogs in a food competition context^43^. Play/comfort calls (N_sound_=10) were recorded during playful interaction (e.g., during tug-of-war) with the owner (play pants, grunts and growls^43,45^ and greeting or petting (pants and grunts^16^. Earlier studies showed that growls^43^ and pants^100,101^ emitted during play are acoustically distinct, rhythmic series of short, atonal, turbulent noise-bursts produced through forced in- and exhalation and/or low-pitched, harsh calls and perceived differently from agonistic growls both by dogs^43,45^ and by humans^18^.

In Study 2, for comparability, we aimed to match the contexts and the assumed inner states across the different species as much as possible. The human non-verbal calls were obtained from the extended sound corpus of Anikin and Persson^46^. These are natural, non-acted recordings from different adults (half of the selected pool was female, the other half male). The human speech set was collected from the RAVDESS corpus^47^ consisting of 3–4 s-long sections of English sentences with acted intonations. The chimpanzee calls were collected by one of the co-authors (KES)^20^, containing field recordings of the Sonso community of wild chimpanzees in Budongo Forest, Uganda and zoo recordings of captive chimpanzees housed at Edinburgh Zoo and the Wolfgang Kohler Primate Research Centre, Leipzig, Germany. Additional samples were collected from the Gombe database^48^, and obtained via Macaulay Library.

For distress calls (N_sound_=12) in the human non-verbal group, we chose adult cries linked with sadness evoked by loss or grieving from contexts of mourning or breakups^46^. While human baby cries evoke less attention from dogs^102^, pretended adult cries were reported to evoke empathy-like reactions from them^103^. In the case of chimpanzee distress calls (N_sound_=14), we collected separation whimpers of young individuals. These whimpers are used by young and adolescent individuals when separated from their mothers, but not used by adults^104^; thus, they can be considered as contact vocalizations similar to human infant separation calls and functionally analogous with adult dog separation whines. For agonistic human non-verbal vocalizations (N_sound_=32), we selected rage roars and screams linked with anger, recorded in contexts such as losing in a game, detecting a cheater or facing hindrance or malfunction^46^. Such rage roars communicate physical strength and body height^105,106^, function to intimidate others, and have acoustic parallels with chimpanzee threatening calls^107^. Therefore, we chose waa-barks (N_sound_=35) from chimpanzees used in agonistic conflicts^20,107^. These calls are short but intense, with a dome-shaped pitch contour emitted once or in short sequences, and mainly function to repel an aggressive individual or predator^20^, although they are also considered as mobbing/recruitment calls^108^. Play/comfort human non-verbal calls (N_sound_=16) were amusement laughs collected from situations such as social play or funny contexts (e.g. pranks, failed stunts)^46^. Human laughter is considered a play vocalization^109^, phylogenetically linked to primate play vocalizations^110^. Thus, we selected chimpanzee laughs (N_sound_=13) for our playbacks recorded during tickling and play wrestling^20^. Both human and chimpanzee laughs serve an interaction-maintaining function^111^ and can evoke a positive emotional state from others through emotional contagion^112^.

Speech samples were acted recordings of two English sentences (“Kids are talking at the door” or “Dogs are sitting at the door”), carrying no linguistic meaning for the participating dogs. Their intonation reflected sad (N_sound_=12), angry (N_sound_=14) or happy (N_sound_=12) emotional states validated in playback tests to match our chosen distress, agonistic and play/comfort contexts, respectively^47^.

We chose samples containing at least one clean call emitted by one individual and with as low background noise as possible. Selected sound samples were converted to mono and to have the same sampling rate (44.1 kHz) when necessary, then RMS normalized to the same level (−63dB). Then, we used a custom Praat script to assemble 60 ± 2 s long sequences of various individuals’ sounds. One sequence contained all samples from the same context (distress, agonistic, play/comfort) and the same calling species/type (dog, chimpanzee, human non-verbal, speech) in randomized order, and samples were separated by shorter (0.5–1.5 s) or longer pauses (2–3 s) in semi-random order. The break lengths were chosen for the particular subgroup of sessions based on the samples’ lengths to ensure the sequences’ final length. We chose these assembly rules to ensure variability in the call presentation while avoiding pseudo-replication and possible quick habituation to a regularly timed sequence. From the numerous possible orders (over 3.5 million possible ones, just in case of the dog calls), we generated 44 unique sequences for each group and each participant was assigned one at random. Each unique sequence was used only once for playback.

### Set up

We performed the playbacks in a laboratory room of the Department of Ethology, ELTE. The lab is 5 × 3 m long; the main, soundproof door opens onto a corridor along the midline of one of the shorter walls. The left-hand wall has a soundproof door on the far end to the neighboring observing room from which the experimenter controlled the playbacks and followed the events in the lab through a closed-circuit camera system (three Logitech USB web cameras) with a Zoom H4n USB sound card equipped with Sennheiser ME-62 omnidirectional microphone and K6 power module for sound, and this equipment was also used to record the tests. The right wall is a mobile wooden/plastic wall with a door on the far end separating the small lab from a larger empty compartment during the tests. A single speaker (Technics SB-M300M2, 40–45,000 Hz, 60 W, 6 Ohm, 85 dB) was used for the playbacks, linked to Technics preamplifier (se-a909s) and a power amplifier (su-c909u) connected to a desktop PC. During the tests, subjects used only the main door from the corridor to enter and exit the lab and had no experience with the other doors.

**Fig. 6:**
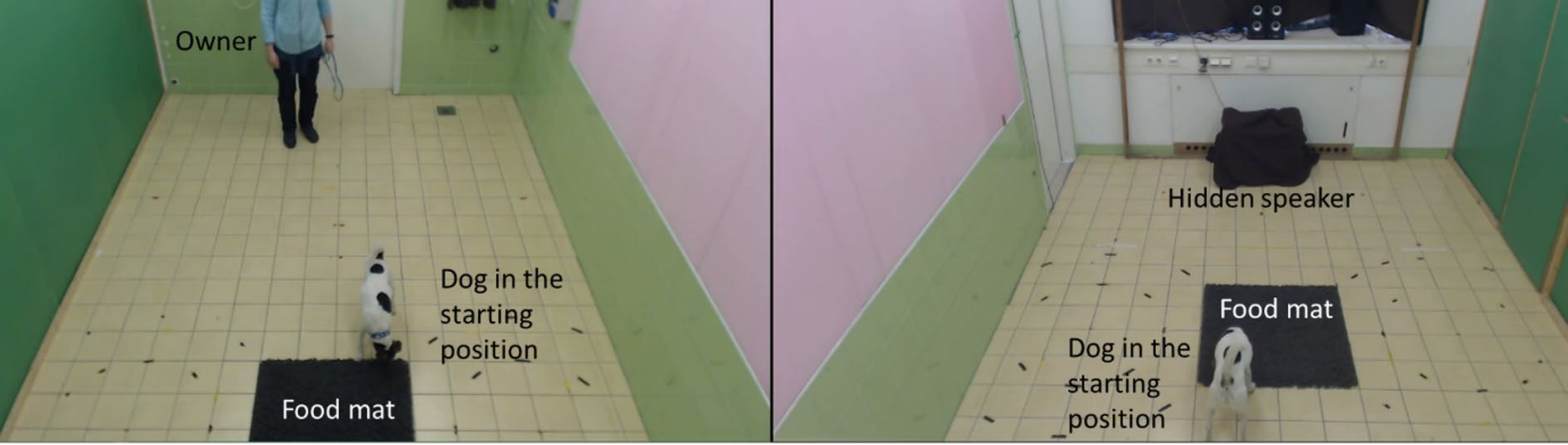
The lab set-up.

The lab arrangement was a modified version of Faragó et al. (2010)^43^(Fig. 6). Opposite the main door, we placed a blanket-covered plastic crate containing the speaker in the midline. A food mat was placed on the floor from the box at 120 centimeters (75 × 80 cm).

### Procedure

The experiment consisted of three phases.

1. *Familiarization/warm-up phase*.

We hid five treats on the carpet (dry pellets or sausage). If the dogs had a special diet or allergies, we asked the owners to bring their own treats to use. The experimenter opened the lab door so the owner and the dog could enter the room. Then, they were asked to walk around the room along the walls with the dog on a leash, letting the dog sniff the treats on the mat without letting it eat them. When they arrived at the main door again, the owner let the dog off the leash, encouraged it to go to the mat, and allowed it to search the carpet and eat the treats. When all treats were found, the phase ended. With this phase, we let the dogs explore the room and ensured that in the test phase, they would be motivated to go to the mat; thus, all dogs were in roughly the same position and distance from the speaker when the playback started.

*2. Break*.

After the Familiarization/warm-up phase, there was a 3-minute break. The experimenter led the owner and dog through the corridor to the neighboring compartment. The owner sat in a chair in the middle of the compartment while the dog was let off the leash, free to explore the room. The owner was allowed to pet their dogs when s/he came to them.

*3. Test phase*.

The experimenter asked the owner to put the dog on a leash and led them back to the small lab room while ensuring the owner was familiar with the instructions. Upon entering the room, the owner had to stand at the door. After the door was closed, they waited 10 s, then let the dog off leash and either stood passively or, if the dog did not move, encouraged it to go to the mat or gave a releasing command if the dog was trained to avoid food on the floor. The experimenter used the 10-second pause to return to the preparation room to start the playback at the appropriate moment. The experimenter started the playback when the dog contacted the food mat with at least one leg. The owner was asked to remain passive and neutral without speaking to or interacting with the dog. The one-minute playback ran without interruption, and the dog was allowed to behave and react freely. The end of the playback marked the end of the phase.

### Data analysis

We coded three possible initial reactions from the video recordings and measured their latencies from the start to the end of the playback (~ 60 s).

Initial reactions:

- Approach reaction was coded when the dog moved from the starting point towards the speaker.
- Withdrawal reaction was coded when the dog moved from the starting point away from the speaker.
- No reaction was coded if the dog stayed on the mat during the whole playback and was censored at 60 s.

### Latencies of initial reactions

Latencies were measured from the start of playback until the initial reaction: in the case of approach latency, when the dog moved from the starting point towards the speaker, while in the case of withdrawal latency, the first movement from the starting point pointing away from the speaker. Those dogs that first showed avoidance were considered censored cases for approach with the latency of their avoidance reaction, and vice versa.

LK (*N* = 153) and TF (*N* = 84) coded most of the videos, while SBR (*N* = 34) and an independent coder (*N* = 24) re-coded two random subsamples of the videos for reliability measures. All coders analyzed the videos blinded to the playback type. This was achieved by muting the videos, while the playback start and end were marked using an oscillogram-type visualization of the soundtrack, and confirmed by sound after the behavior coding. Latency measures were highly correlated across the coders (Robust correlations - Approach: r_w_[95%CI] = 1.00[1.00,1.00], t(22) = 134.43, *p* < 0.001 and r_w_[95%CI] = 0.92[0.84,0.96], t(32) = 13.25, *p* < 0.001; Withdrawal: r_w_[95%CI] = 0.87[0.73,0.94], t(22) = 8.43, *p* < 0.001 and r_w_[95%CI] = 0.81[0.65,0.90], t(32) = 7.88, *p* < 0.001) and thus the coding can be considered reliable.

### Statistics

All statistical tests were performed in R^113^ and RStudio^114^. A detailed list of used packages and analysis scripts can be found in the ESM.

We used the Generalized Linear Model with binomial response family and logit link (glm) to analyze the occurrence of initial reactions (approach or withdrawal, no responses were treated as missing values). For latencies, we applied Cox proportional hazard models (see^53^. Model assumptions were visually confirmed with the check_model function. For model comparisons, we used the compare_performance function (both from the easystats package). The glmulti function was used with an exhaustive search method based on AICc values for model inference. We applied Likelihood Ratio tests (drop1 function) for further model comparisons. For pairwise post-hoc comparisons, we used Tukey tests (emmeans function).

## Supplementary Information

Below is the link to the electronic supplementary material.

Supplementary Material 1

Supplementary Material 2

Supplementary Material 3

Supplementary Material 4

## Data Availability

All raw data is available in the Supplementary Materials. An example of the Praat script used to assemble the playback sequences and the R script used for the statistical analysis can be found in the Supplementary Materials too.
