# Supplementary material for: Dogs’ reactions to motivations and emotions in conspecific and heterospecific vocalizations
Source: Sci Rep. 2026 Apr 1;16:15360. doi: 10.1038/s41598-026-46906-y (PMC13184305; doi:10.1038/s41598-026-46906-y)
Supplement: Supplementary file 3 — Supplementary Material 3 [file 41598_2026_46906_MOESM3_ESM.pdf]

Supplementary Materials

Dogs’ reactions to motivations and emotions in conspecific and heterospecific vocalizations

Tamás Faragó[1,2,*], Lilla Kocsis[3], Beatrix Laczi[1, 3], Irene Rojas Atares[3], Paula Pérez Fraga[1,2], Morgane Audiguier[3], Soufiane Bel Rhali[3], Katie E. Slocombe[4], Enikő Kubinyi[5,6,¶], Attila Andics,[1,6,¶]

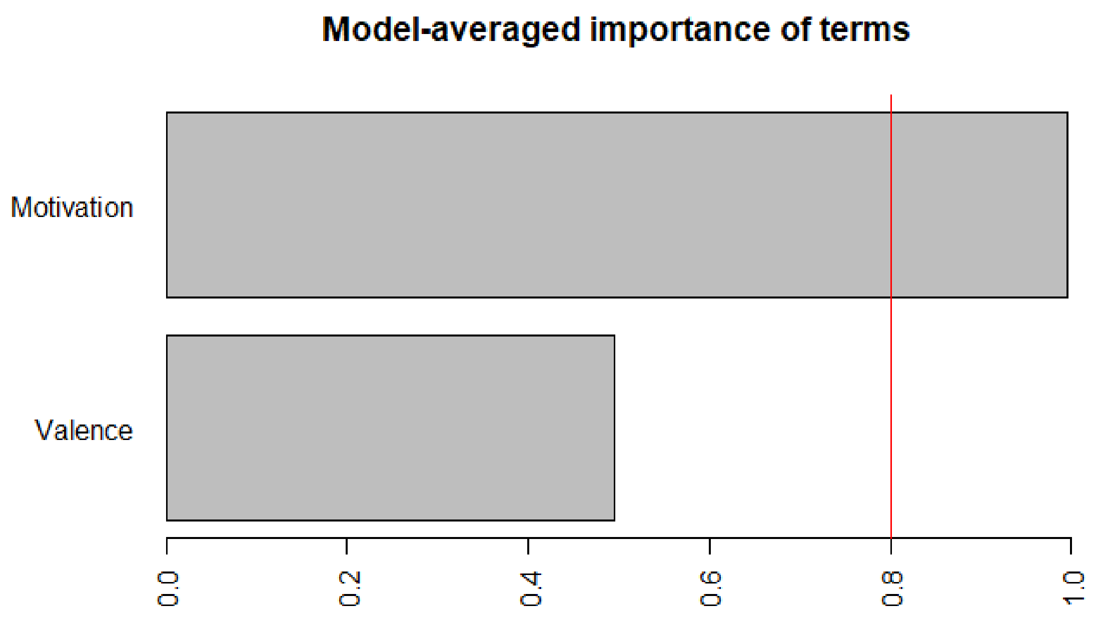

Fig. S1.
Importance of terms derived from binomial model inference and averaging on the initial reactions to conspecific sounds.

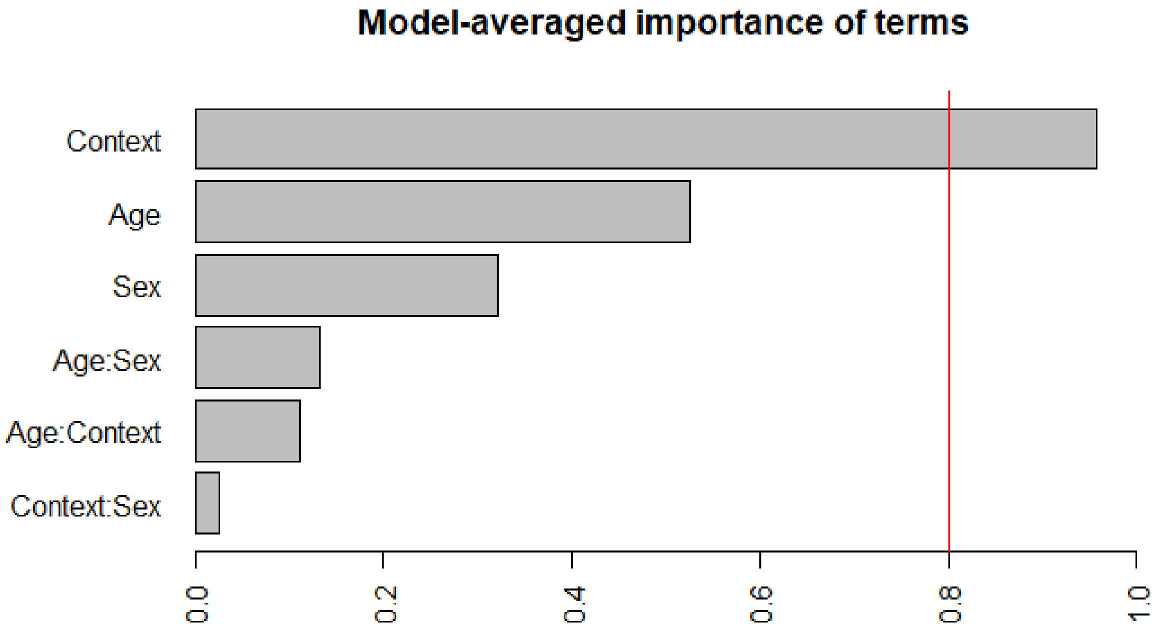

Fig. S2.
Importance of terms derived from cox model inference and averaging on the approach latencies to conspecific sounds.

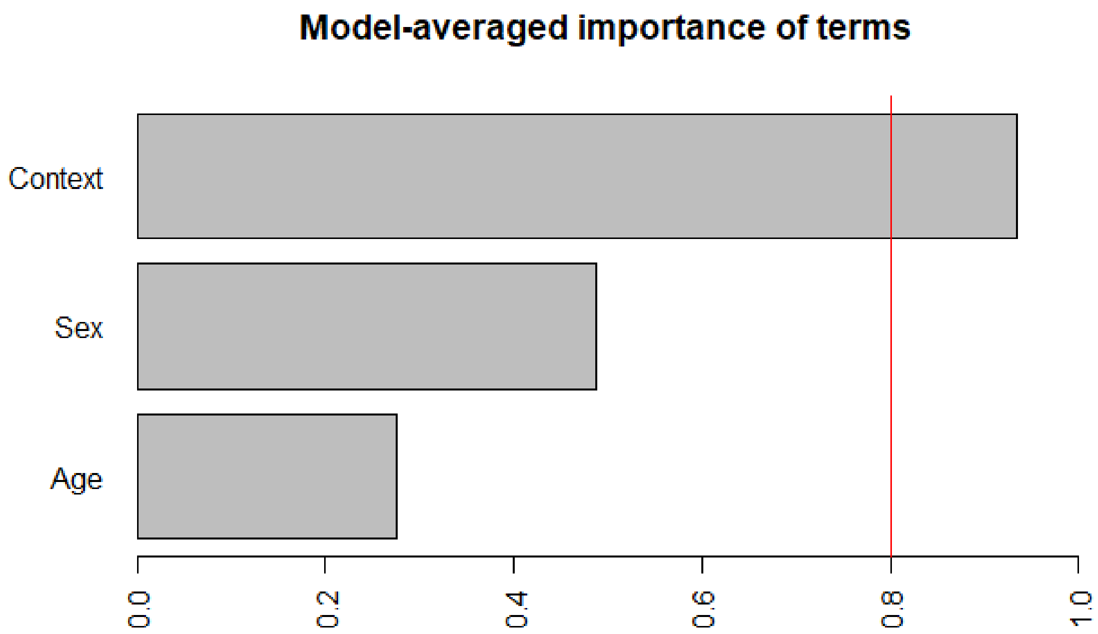

Fig. S3.
Importance of terms derived from cox model inference and averaging on the withdrawal latencies.

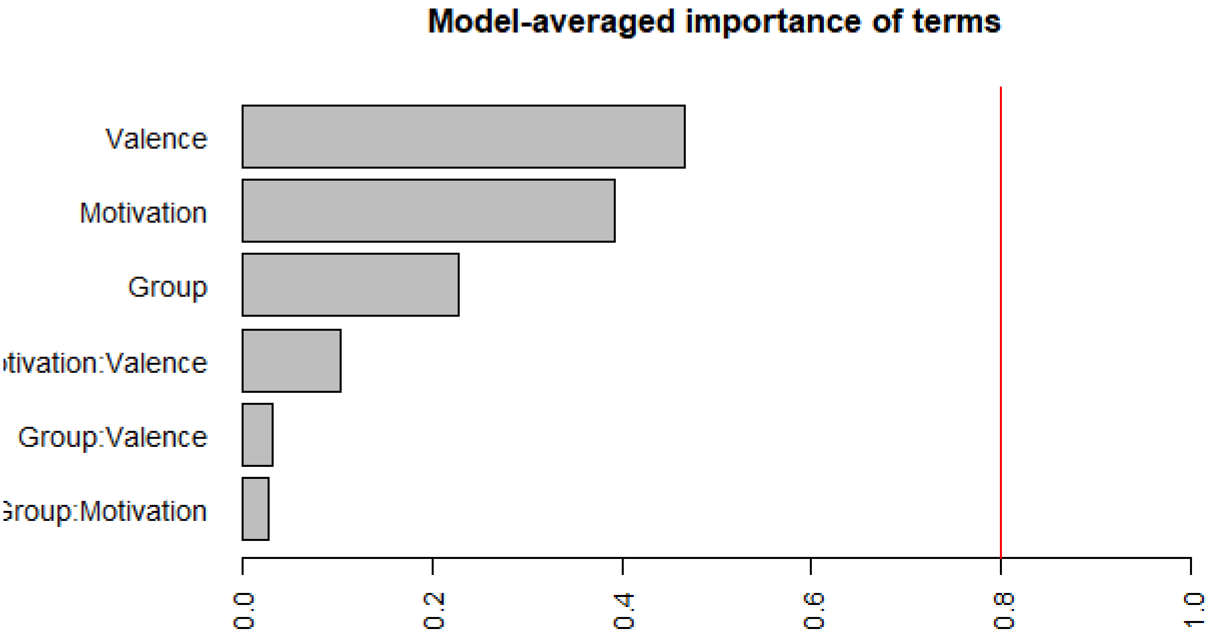

Fig. S4.
Importance of terms derived from binomial model inference and averaging on the initial reactions to heterospecific sounds.

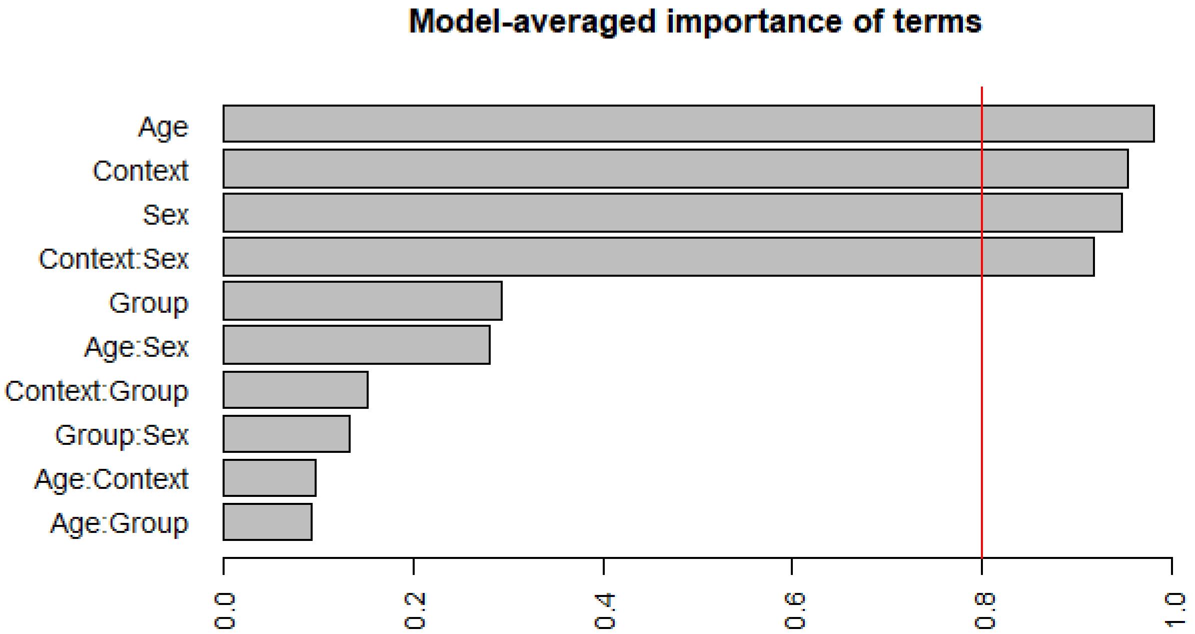

Fig. S5
Importance of terms derived from cox model inference and averaging on the approach latencies to heterospecific sounds.

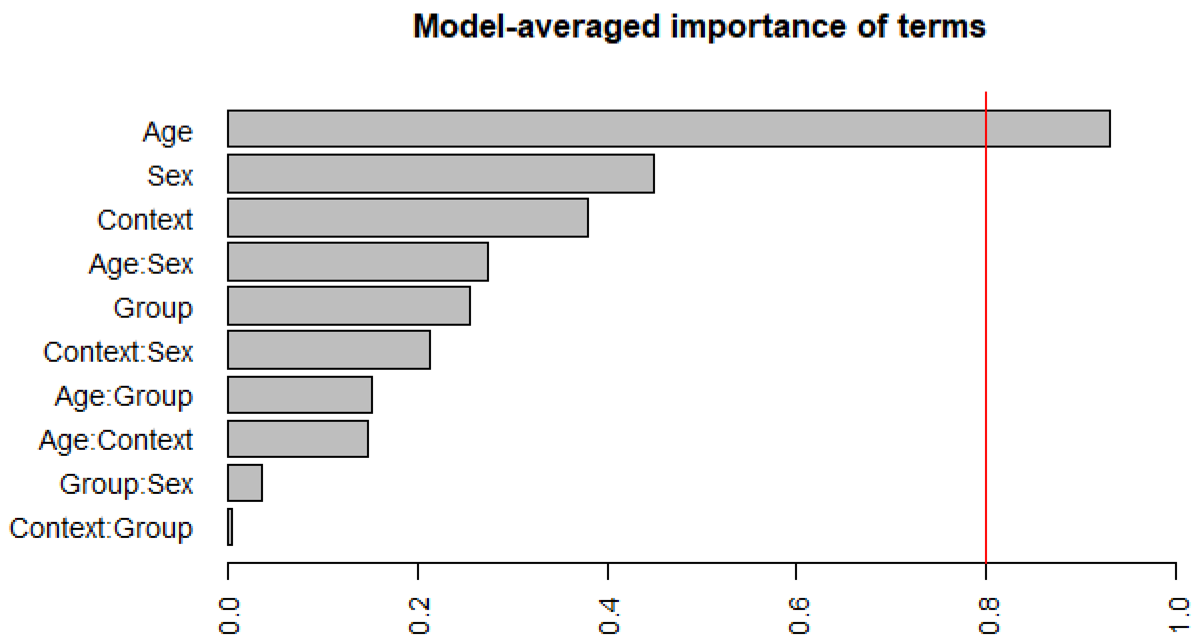

Fig. S6.
Importance of terms derived from cox model inference and averaging on the withdrawal latencies to heterospecific sounds.

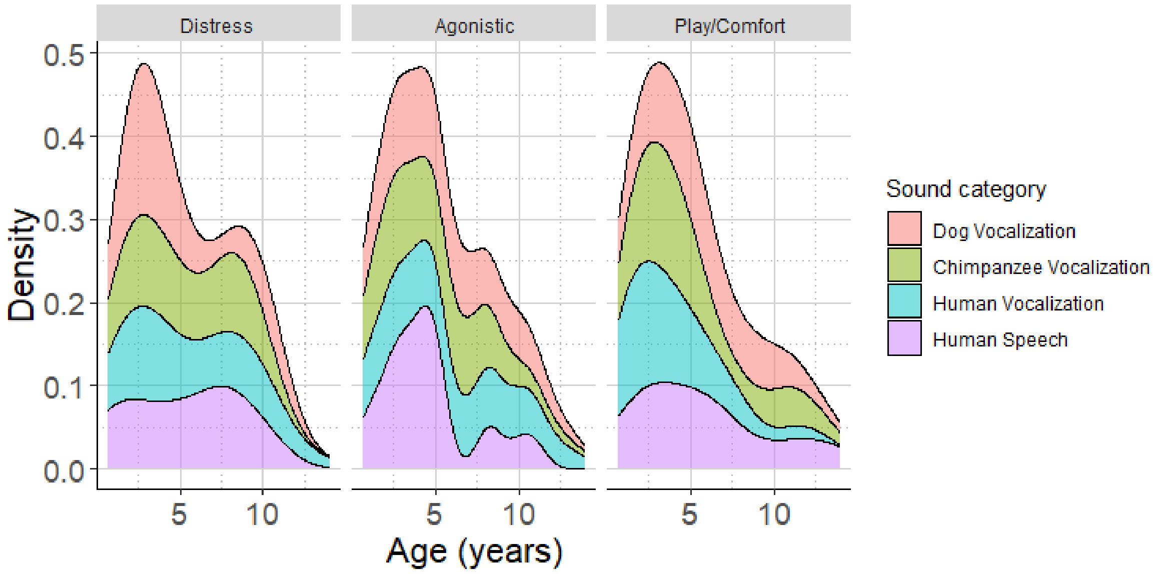

Fig. S7.
Age distribution across the subgroups of Study1 and 2

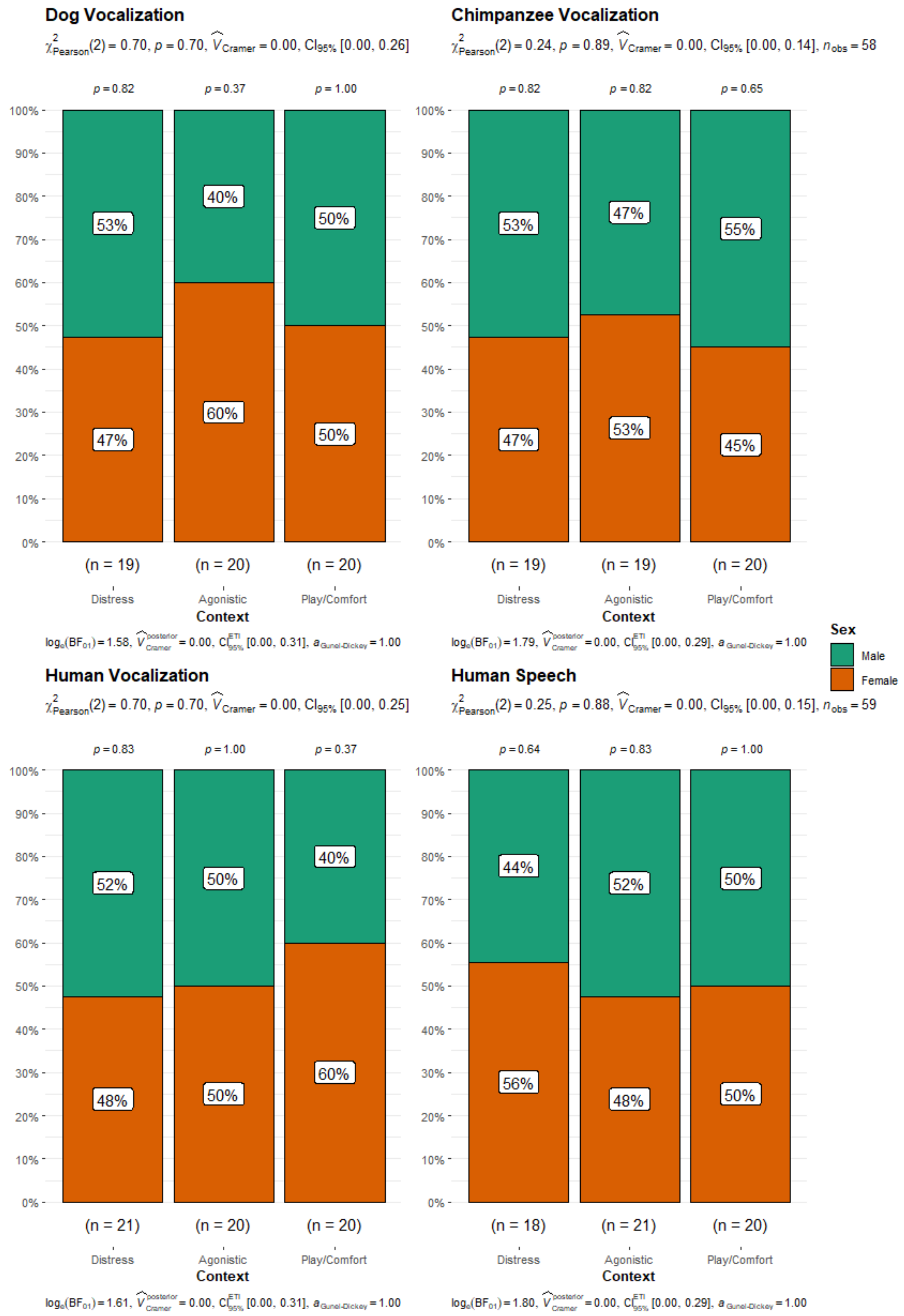

Fig. S8.

Sex distribution across subgroups.

model terms	aicc	weights
~ 1 + Context	288.75	0.30
~ 1 + Context + Age	289.57	0.20
~ 1 + Context + Sex	290.27	0.14
~ 1 + Context + Sex + Age	291.21	0.09
~ 1 + Context + Age + Context:Age	292.08	0.06
~ 1 + Context + Sex + Age + Sex:Age	293.80	0.02
~ 1 + Context + Sex + Age + Context:Age	294.23	0.02
~ 1 + Context + Sex + Sex:Context	294.82	0.01
~ Intercept	295.83	0.01
~ 1 + Context + Sex + Age + Sex:Context	296.03	0.01
~ 1 + Sex	296.16	0.01
~ 1 + Age	296.91	0.01
~ 1 + Context + Sex + Age + Context:Age + Sex:Age	297.10	0.00
~ 1 + Sex + Age	297.12	0.00
~ 1 + Context + Sex + Age + Sex:Context + Sex:Age	298.95	0.00
~ 1 + Sex + Age + Sex:Age	299.24	0.00
~ 1 + Context + Sex + Age + Sex:Context + Context:Age	299.87	0.00
~ 1 + Context + Sex + Age + Sex:Context + Context:Age + Sex:Age	303.00	0.00

Table S1.

The results of the model inference for the within-species cox models for the approach latencies.

model terms	aic	weights
~ 1 + Context	111.03	0.36
~ 1 + Context + Sex	111.21	0.32
~ 1 + Context + Age	113.00	0.13
~ 1 + Context + Sex + Age	113.16	0.12
~ 1 + Sex	116.07	0.03
~ Intercept	117.20	0.02
~ 1 + Sex + Age	117.71	0.01
~ 1 + Age	118.74	0.01

Table S2.
The results of the model inference for the within-species cox models for the withdrawal latencies.

model terms	aicc	weights
~ 1 + Context + Sex + Age + Sex:Context	1138.43	0.36
~ 1 + Context + Sex + Age + Sex:Context + Sex:Age	1140.13	0.15
~ 1 + Group + Context + Sex + Age + Context:Group + Sex:Context	1142.42	0.05
~ 1 + Group + Context + Sex + Age + Context:Group + Sex:Group + Sex:Context	1142.48	0.05
~ 1 + Group + Context + Sex + Age + Sex:Context	1142.68	0.04
~ 1 + Context + Sex + Age + Sex:Context + Context:Age	1142.95	0.04
~ 1 + Group + Context + Sex + Age + Sex:Group + Sex:Context	1143.15	0.03
~ 1 + Group + Context + Sex + Age + Sex:Context + Sex:Age	1144.37	0.02
~ 1 + Context + Sex + Age + Sex:Context + Context:Age + Sex:Age	1144.69	0.02
~ 1 + Group + Context + Sex + Age + Context:Group + Sex:Context + Sex:Age	1144.71	0.02
~ 1 + Group + Context + Sex + Age + Context:Group + Sex:Group + Sex:Context + Sex:Age	1144.95	0.01
~ 1 + Group + Context + Sex + Age + Sex:Group + Sex:Context + Sex:Age	1145.00	0.01
~ 1 + Context + Sex + Sex:Context	1145.22	0.01
~ 1 + Age	1145.33	0.01
~ 1 + Context + Age	1145.37	0.01
~ 1 + Context + Sex + Age	1146.64	0.01
~ 1 + Sex + Age	1146.77	0.01

Table S3.

The results of the model inference for the cross-species cox models for the approach latencies. Models with weight smaller than 0.01 are omitted.

	Approach_latency			
Predictors	*Estimates*	*CI*	*Statistic*	*p*
Age	0.923	0.874 – 0.974	-2.907	**0.004**
Distress	*Reference*			
ContextAgonistic:SexMale	4.635	1.957 – 10.977	3.487	**<0.001**
Agonistic	0.535	0.290 – 0.986	-2.005	**0.045**
Play/Comfort	0.535	0.292 – 0.979	-2.029	**0.043**
ContextPlay/Comfort:SexMale	1.808	0.743 – 4.399	1.306	0.191
Female	*Reference*			
Male	0.577	0.313 – 1.064	-1.762	0.078
Observations	178			
R^2 Nagelkerke	0.130			

Table S4.

Summary of the parsimonious cox regression for approach latencies to heterospecific sounds

Model terms	aic	weights
~ 1 + Age	483.35	0.15
~ 1 + Context + Sex + Age + Sex:Context	484.83	0.07
~ 1 + Sex + Age	485.29	0.06
~ 1 + Group + Age	485.70	0.05
~ 1 + Context + Age	485.73	0.05
~ 1 + Context + Sex + Age + Sex:Context + Sex:Age	486.55	0.03
~ 1 + Sex + Age + Sex:Age	486.93	0.02
~ Intercept	487.01	0.02
~ 1 + Group + Context + Sex + Age + Sex:Context	487.34	0.02
~ 1 + Context + Sex + Age	487.65	0.02
~ 1 + Group + Sex + Age	487.69	0.02
~ 1 + Group + Context + Age	488.28	0.01
~ 1 + Sex + Age + Group:Age	488.29	0.01
~ 1 + Group + Context + Sex + Age + Sex:Context + Sex:Age	488.64	0.01
~ 1 + Context + Sex + Age + Sex:Context + Context:Age	488.69	0.01
~ 1 + Sex	488.94	0.01
~ 1 + Group + Sex + Age + Sex:Age	488.98	0.01
~ 1 + Group + Context + Sex + Age + Sex:Group + Sex:Context	489.18	0.01
~ 1 + Context + Sex + Sex:Context	489.18	0.01
~ 1 + Context + Sex + Age + Sex:Age	489.25	0.01
~ 1 + Group	489.25	0.01
~ 1 + Group + Sex + Age + Sex:Group	489.46	0.01
~ 1 + Context	489.60	0.01
~ 1 + Group + Age + Group:Age	489.63	0.01
~ 1 + Context + Age + Context:Age	489.67	0.01

Table S5.

The results of the model inference for the cross-species cox models for the withdrawal latencies. Models with weight smaller than 0.01 are omitted.

	**Withdrawal_latency**			
Predictors	*Estimates*	*CI*	*Statistic*	*p*
Age	1.103	1.019 – 1.194	2.419	**0.016**
Observations	178			
R^2 Nagelkerke	0.033			

Table S6.

Summary of the parsimonious cox regression for withdrawal latencies to heterospecific sounds

Predictors	*Estimates*	*CI*	*Statistic*	*p*
(Intercept)	4.700	3.209 – 6.191	6.212	**<0.001**
Group [Chimpanzee Vocalization]	0.537	-1.572 – 2.645	0.502	0.616
Group [Human Vocalization]	0.800	-1.258 – 2.858	0.766	0.444
Group [Human Speech]	0.572	-1.565 – 2.710	0.528	0.598
Context [Agonistic]	0.805	-1.277 – 2.887	0.762	0.447
Context [Play/Comfort]	0.940	-1.142 – 3.022	0.890	0.375
Group [Chimpanzee Vocalization] X Context [Agonistic]	-1.126	-4.089 – 1.837	-0.749	0.455
Group [Human Vocalization] X Context [Agonistic]	-0.690	-3.598 – 2.218	-0.468	0.641
Group [Human Speech] X Context [Agonistic]	-1.515	-4.464 – 1.433	-1.013	0.312
Group [Chimpanzee Vocalization] X Context [Play/Comfort]	-1.022	-3.966 – 1.923	-0.684	0.495
Group [Human Vocalization] X Context [Play/Comfort]	-2.705	-5.613 – 0.203	-1.833	0.068
Group [Human Speech] X Context [Play/Comfort]	-0.487	-3.453 – 2.478	-0.324	0.746
Observations	237			
R^2 / R^2 adjusted	0.029 / -0.018			

Table S7.
Age differences across subgroups

Parameter	mot_mod	mot_val_mod	null_mod	val_mod
(Intercept)	0.73 (0.29, 1.81)	0.73 (0.29, 1.81)	2.56** (1.44, 4.57)	2.17* (1.09, 4.29)
Motivation [Non-hostile]	9.07*** (2.45, 33.61)	24.75** (2.72, 225.61)		
Valence [Positive]		0.21 (0.02, 2.07)		1.73 (0.47, 6.34)
Observations	57	57	57	57

Table S8

The parameter details of all tested binomial models in Study 1

Parameter	appr_vi
(Intercept)	3.75*** (1.72, 8.18)
Valence [Positive]	0.30 (0.09, 1.03)
Group [Human Vocalization]	0.80 (0.28, 2.31)
Group [Human Speech]	0.53 (0.19, 1.49)
Group [Human Speech] × Valence [Positive]	3.10 (0.58, 16.51)
Group [Human Vocalization] × Valence [Positive]	3.33 (0.58, 19.12)
Motivation [Non-hostile]	
Group [Human Speech] × Motivation [Non-hostile]	
Group [Human Vocalization] × Motivation [Non-hostile]	
Observations	174

Parameter	appr_si
(Intercept)	5.33** (1.55, 18.30)
Valence [Positive]	
Group [Human Vocalization]	0.70 (0.13, 3.68)
Group [Human Speech]	0.30 (0.07, 1.39)
Group [Human Speech] × Valence [Positive]	
Group [Human Vocalization] × Valence [Positive]	
Motivation [Non-hostile]	0.33 (0.08, 1.36)
Group [Human Speech] × Motivation [Non-hostile]	4.02 (0.67, 24.23)
Group [Human Vocalization] × Motivation [Non-hostile]	2.19 (0.32, 14.92)
Observations	174

Parameter	appr_s_vi
(Intercept)	4.01** (1.65, 9.79)
Valence [Positive]	0.32 (0.09, 1.17)
Group [Human Vocalization]	0.80 (0.28, 2.31)
Group [Human Speech]	0.53 (0.19, 1.48)
Group [Human Speech] × Valence [Positive]	3.11 (0.58, 16.62)
Group [Human Vocalization] × Valence [Positive]	3.32 (0.58, 19.07)
Motivation [Non-hostile]	0.87 (0.38, 2.00)
Group [Human Speech] × Motivation [Non-hostile]	
Group [Human Vocalization] × Motivation [Non-hostile]	
Observations	174

Parameter	appr_v_si
(Intercept)	5.33** (1.55, 18.30)
Valence [Positive]	0.70 (0.32, 1.55)
Group [Human Vocalization]	0.70 (0.13, 3.68)
Group [Human Speech]	0.30 (0.07, 1.39)
Group [Human Speech] × Valence [Positive]	
Group [Human Vocalization] × Valence [Positive]	
Motivation [Non-hostile]	0.39 (0.09, 1.70)
Group [Human Speech] × Motivation [Non-hostile]	4.10 (0.68, 24.80)
Group [Human Vocalization] × Motivation [Non-hostile]	2.21 (0.32, 15.07)
Observations	174

Parameter	appr_gv
(Intercept)	2.78** (1.48, 5.22)
Valence [Positive]	0.67 (0.34, 1.32)
Group [Human Vocalization]	1.25 (0.54, 2.85)
Group [Human Speech]	0.81 (0.36, 1.79)
Group [Human Speech] × Valence [Positive]	
Group [Human Vocalization] × Valence [Positive]	
Motivation [Non-hostile]	
Group [Human Speech] × Motivation [Non-hostile]	
Group [Human Vocalization] × Motivation [Non-hostile]	
Observations	174

Parameter	appr_gs

(Intercept)	2.99** (1.40, 6.38)
Valence [Positive]	
Group [Human Vocalization]	1.24 (0.54, 2.84)
Group [Human Speech]	0.80 (0.36, 1.76)
Group [Human Speech] × Valence [Positive]	
Group [Human Vocalization] × Valence [Positive]	
Motivation [Non-hostile]	0.74 (0.36, 1.50)
Group [Human Speech] × Motivation [Non-hostile]	
Group [Human Vocalization] × Motivation [Non-hostile]	
Observations	174

Parameter	appr_full
(Intercept)	5.33** (1.55, 18.30)
Valence [Positive]	0.40 (0.10, 1.62)
Group [Human Vocalization]	0.70 (0.13, 3.68)
Group [Human Speech]	0.30 (0.07, 1.39)
Group [Human Speech] × Valence [Positive]	1.78 (0.25, 12.67)
Group [Human Vocalization] × Valence [Positive]	2.99 (0.42, 21.36)
Motivation [Non-hostile]	0.52 (0.11, 2.60)
Group [Human Speech] × Motivation [Non-hostile]	3.05 (0.37, 24.84)
Group [Human Vocalization] × Motivation [Non-hostile]	1.27 (0.15, 11.04)
Observations	174

Parameter	appr_g
(Intercept)	2.44** (1.36, 4.36)
Valence [Positive]	
Group [Human Vocalization]	1.23 (0.54, 2.81)
Group [Human Speech]	0.80 (0.36, 1.77)
Group [Human Speech] × Valence [Positive]	
Group [Human Vocalization] × Valence [Positive]	
Motivation [Non-hostile]	
Group [Human Speech] × Motivation [Non-hostile]	
Group [Human Vocalization] × Motivation [Non-hostile]	
Observations	174

Parameter	appr_v
(Intercept)	2.77*** (1.84, 4.18)
Valence [Positive]	0.67 (0.34, 1.32)
Group [Human Vocalization]	
Group [Human Speech]	
Group [Human Speech] × Valence [Positive]	
Group [Human Vocalization] × Valence [Positive]	
Motivation [Non-hostile]	
Group [Human Speech] × Motivation [Non-hostile]	
Group [Human Vocalization] × Motivation [Non-hostile]	
Observations	174

Parameter	appr_s
(Intercept)	2.93*** (1.63, 5.27)
Valence [Positive]	
Group [Human Vocalization]	
Group [Human Speech]	
Group [Human Speech] × Valence [Positive]	
Group [Human Vocalization] × Valence [Positive]	
Motivation [Non-hostile]	0.75 (0.37, 1.52)
Group [Human Speech] × Motivation [Non-hostile]	
Group [Human Vocalization] × Motivation [Non-hostile]	
Observations	174

Parameter	appr_s_v
(Intercept)	2.93*** (1.63, 5.27)
Valence [Positive]	0.70 (0.32, 1.56)

Group [Human Vocalization]	
Group [Human Speech]	
Group [Human Speech] × Valence [Positive]	
Group [Human Vocalization] × Valence [Positive]	
Motivation [Non-hostile]	0.89 (0.39, 2.04)
Group [Human Speech] × Motivation [Non-hostile]	
Group [Human Vocalization] × Motivation [Non-hostile]	
Observations	174

Table S9

The parameter details of all tested binomial models in Study 2
